# Single, Double and Triple Blockade of RAAS in Alport Syndrome: Different Tools to Freeze the Evolution of the Disease

**DOI:** 10.3390/jcm10214946

**Published:** 2021-10-26

**Authors:** Antonio Mastrangelo, Marta Brambilla, Giorgia Romano, Jessica Serafinelli, Giuseppe Puccio, Marisa Giani, Giovanni Montini

**Affiliations:** 1 Pediatric Nephrology, Dialysis and Transplant Unit, Fondazione IRCCS Ca’ Granda Ospedale Maggiore Policlinico, 20122 Milan, Italy; marta.jua.brambilla@gmail.com (M.B.); Jessica.serafinelli@policlinico.mi.it (J.S.); Marisa.giani@policlinico.mi.it (M.G.); giovanni.montini@unimi.it (G.M.); 2 Pediatric Unit, University Hospital of Udine, 33100 Udine, Italy; romano.giorgia86@gmail.com; 3 Department of Sciences for Health Promotion, University of Palermo, 90121 Palermo, Italy; gipuccio@gmail.com; 4 Department of Clinical Sciences and Community Health, University of Milan, 20122 Milan, Italy

**Keywords:** Alport syndrome, proteinuria, RAAS, spironolactone, angiotensin-converting enzyme inhibition, angiotensin receptor blockade

## Abstract

Background: The goal of the treatment of Alport syndrome (AS) is to delay the progression of kidney damage. The current standard of care is the use of Renin Angiotensin Aldosterone System (RAAS) blockers: angiotensin-converting enzyme inhibition (ACEi), angiotensin receptor blockade, and, recently, spironolactone (SP). Aim of the study: the purpose of this retrospective study is to evaluate the efficacy (reduction of proteinuria and changes of glomerular function) and safety of a sequential introduction of RAAS blockers up to a triple RAAS blockade in pediatric proteinuric patients with AS. Methods: in this retrospective study (1995 to 2019), we evaluated proteinuria values in AS patients, during the 12 months following the beginning of a new RAAS blocker, up to a triple blockade. ACEi was always the first line of treatment; then ARB and SP were sequentially added if uPCR increased by 50% from the basal level in 2 consecutive samples during a 3-months observation period, or when uPCR ratio was >2 mg/mg. Results: 26 patients (mean age at treatment onset was 10.55 ± 5.02 years) were enrolled. All patients were on ACEi, 14/26 were started on a second drug (6/14 ARB, 8/14 SP) after a mean time of 2.2 ± 1.7 years, 7/26 were on triple RAAS blockade after a further period of 5.5 ± 2.3 years from the introduction of a second drug. Repeated Measure Anova analysis of log-transformed data shows that the reduction of uPCR values after Time 0 from the introduction of the first, second and third drug is highly significant in all three cases (*p* values = 0.0016, 0.003, and 0.014, respectively). No significant changes in eGFR were recorded in any group, apart from a 15-year-old boy with X-linked AS, who developed kidney failure. One patient developed mild hyperkaliemia, and one gynecomastia and symptomatic hypotension. No life-threatening events were recorded. Conclusions: double and triple RAAS blockade is an effective and safe strategy to reduce proteinuria in children with AS. Nevertheless, we suggest monitoring eGFR and Kaliemia during follow-up.

## 1. Introduction

Alport syndrome (AS) is a rare, progressive hereditary kidney disease, often associated with sensorineural hearing loss and ocular manifestation, potentially leading to kidney failure (KF). The prevalence of AS is 1 case per 5000. It represents 0.5% of newly developed KF cases in adults [1] and 13% in children [2].

AS is caused by pathogenic variants in the COL4A3, COL4A4, and COL4A5 genes encoding α3, α4, and α5 chains, respectively, of type IV collagen [2,3]. These chains constitute the glomerular basement membrane (GBM) and corresponding pathogenetic mutations interfere with the correct assembly of the α3/α4/α5 (IV) collagen network in the GBM and arrest the developmental switch from the embryonic α1/α1/α2 (IV) network to the mature α3/α4/α5 (IV) network, causing the persistence of an immature GBM. The resulting GBM is more porous, susceptible to endoproteolysis, and it is thought to be more vulnerable to increased (or even normal) filtration pressure. Therefore, thickening and splitting of the GBM in AS kidneys could be considered as a stress response of the podocytes, that lead to a higher secretion of profibrotic chemokines and cytokines in the primary urine that is re-absorbed by the tubular cells, causing tubular scar tissue formation that finally demolishes the kidney. [4] Since progressive renal fibrosis lead to KF, the amount of tubulointerstitial fibrosis is the most accurate histological prognostic factor regarding the evaluation of kidney function. Though some studies are developing new therapeutic strategies, to date, there is no etiological therapy, and no therapeutic option has been definitely shown to prevent the development of terminal renal failure in people with AS. Therefore, the goal of the available pharmacological intervention is to delay the time to dialysis and renal transplantation.

Based on the pathophysiology of AS, any medication that can reduce the intraglomerular blood pressure, such as Renin-Angiotensin-Aldosterone System (RAAS) blockers, is considered to be able to prevent the mechanical stress on the podocyte and the risk of GBM ruptures that could potentially lead to the development of proteinuria and glomerular sclerosis [5]. RAAS blockers include Angiotensin-Converting Enzyme inhibitors (ACEi), Angiotensin receptor blockers [6], and Aldosterone receptor antagonists such as Spironolactone (SP). Clinical data show that a single or double blockade of RAAS reduces the amount of proteinuria in AS patients [7,8,9]. Recently, it has been suggested that SP is also useful in controlling proteinuria, when used in single or multi-drug therapy [10,11,12,13].

The purpose of this retrospective study is to evaluate the efficacy (reduction of proteinuria and changes of glomerular function) and safety of single, double, and triple blockade of RAAS, after a sequential addiction of ACEi, ARB, and/or SP in pediatric proteinuric patients with AS.

## 2. Materials and Methods

### 2.1. Study Population

We recruited patients among those followed between January 1995 and December 2019 in the outpatient clinics of the Pediatric Nephrology, Dialysis, and Transplant Unit of Fondazione IRCCS Ca’ Granda Ospedale Maggiore Policlinico in Milan.

The inclusion criteria were:

- Patients with a diagnosis of AS based on the presence of persistent glomerular hematuria associated with at least 2 of the following:
  - One or more pathogenic variants in COL4A3, COL4A4, and/or COL4A5 genes;
  - Kidney biopsy suggestive for AS: electron microscopy showing lamellated GBM and/or thinning and thickening of GBM and/or basket weave lesions and/or associated glomerular sclerosis;
  - Family history for AS;
  - Sensory-neural hearing loss;
- Age at onset of treatment <18 years;
- Presence of proteinuria (*see Methods section*);
- Follow-up greater than 12 months from the introduction of each RAAS blocker.

The exclusion criteria were (a) uncertain diagnosis of AS (e.g., electron microscopic study ongoing at the moment of data collection or absence of microscopic hematuria tested every 3 months on 4 consecutive evaluations); (b) follow-up shorter than 12 months after the introduction of each ACEi; (c) presence of other superimposed glomerular diseases and/or dysplastic kidneys and/or associated systemic diseases.

### 2.2. Methods

We have retrospectively analyzed clinical and biochemical data regarding patients with AS, treated with RAAS blockers (1, 2 or 3 drugs) at our department between 1995 and 2019. ACEi (ramipril 6 mg/sm/day or enalapril 12 mg/sm/day) has been the first line of treatment. Therapy was started in our patients at different values of proteinuria before 2000, between 2000 and 2012, and after 2012 according to the expert clinical recommendations published in those different periods (*see results section*). The second drug was added to the treatment (ARB, usually Irbesartan 20 mg/sm/day or SP 12.5 mg/day in patients younger than or equal to 12 years of age, and 25 mg in those older than 12 years) if urine Protein-to-Creatinine ratio (uPCR) increased by 50% from the basal level in 2 consecutive samples or when uPCR ratio was >2 mg/mg, during a 3-months observation period. A further subgroup of patients was then started on a third RAAS blocker.

The following data were collected at the visit before starting each RAAS blocker and then at 1, 3, and 12 months of follow-up: age, treatment, serum potassium, serum creatinine, estimated glomerular filtration rate (eGFR), proteinuria, urine creatinine, blood pressure, and the appearance of cough, headache, liver dysfunction, gynecomastia, allergic reactions, cardiac arrhythmia, and muscle weakness or other side effects.

Hyperkaliemia was defined as serum potassium values over 5.5 mmol/L. Serum creatinine was measured with the Jaffé method, and eGFR was calculated by the Schwartz formula [13]. Proteinuria was expressed as spot uPCR mg/mg [14,15].

Blood pressure was measured using an automatic sphygmomanometer (oscillometric method).

Genetic analysis was performed by locus-specific amplification followed by massively parallel sequencing (454 Junior sequencing Roche, Basel, Switzerland). The mutations identified in probands were confirmed by direct Sanger sequencing and defined to be pathogenetic if already described in the literature or after comparison with the ClinVar, ARUP, or LOVD databases. Patients followed before 2008 often received only a partial genetic analysis. Anyway, our recent management protocol (after 2017) included the need to perform a complete analysis with massively parallel sequencing for all our patients, even in those who had already received a partial test.

#### Statistical Analysis

Data were presented as mean ± standard deviation (SD) and median. The differences in values at different time points after the introduction of each new RAAS blocker were evaluated using Repeated Measures Anova. When data showed a non-normal (right-skewed) distribution (as in the case of uPCR and eGFR) a log transformation was applied before the analysis.

Pairwise comparisons were performed using a paired t test (for normal data) or paired Wilcoxon test (for non-normal data).

For all analyses, a *p* value < 0.05 was considered to be statistically significant.

All statistical analyses were performed using the open-source software R:

R Core Team (2021). R: A language and environment for statistical computing. R Foundation for Statistical Computing, Vienna, Austria. URL https://www.R-project.org/, last accessed on 15 June 2021.

## 3. Results

### 3.1. Patient Population

**Figure 1:**
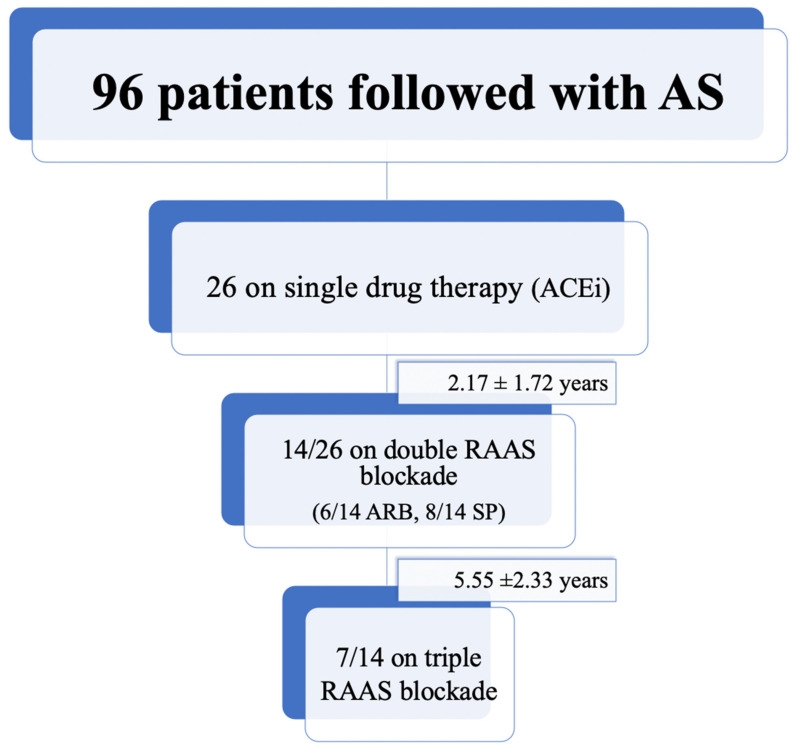
Flowchart relating to patients recruited from the whole cohort and group configuration.

Twenty-six patients (16 females, 61.5%) met the inclusion criteria and were recruited between 1995 and 2019 (Figure 1). Table 1 summarizes demographic, genetic, and clinical data of the patients at baseline.

RAAS treatment was started when uPCR ratio was higher than 1 mg/mg in two consecutive controls during a 3-month observation period in patients treated before 2000 (2 patients), over 0.5 mg/mg in those treated from 2000 to 2012 (12 patients), and over 0.3 mg/mg in patients treated after 2012 (12 patients), according to the expert clinical recommendations published in 2000 by Hogg et al. [14] and to the results from the randomized, prospective, placebo-controlled EARLY PROTECT clinical trial [15].

All patients received at least one RAAS blocker at the time of recruitment. In particular, 26/26 patients were on ACEi (single RAAS blockade), 14/26 (53.8%) were also on ARB (6/14) or SP (8/14), and 7/26 (26.9%) were on triple RAAS blockade

The mean age of patients at treatment onset was 10.55 ± 5.02 years.

Second and third drugs were introduced, respectively, at 2.17 ± 1.72 years and 5.55 ± 2.33 years from the beginning of the therapy.

### 3.2. Proteinuria

**Figure 2:**
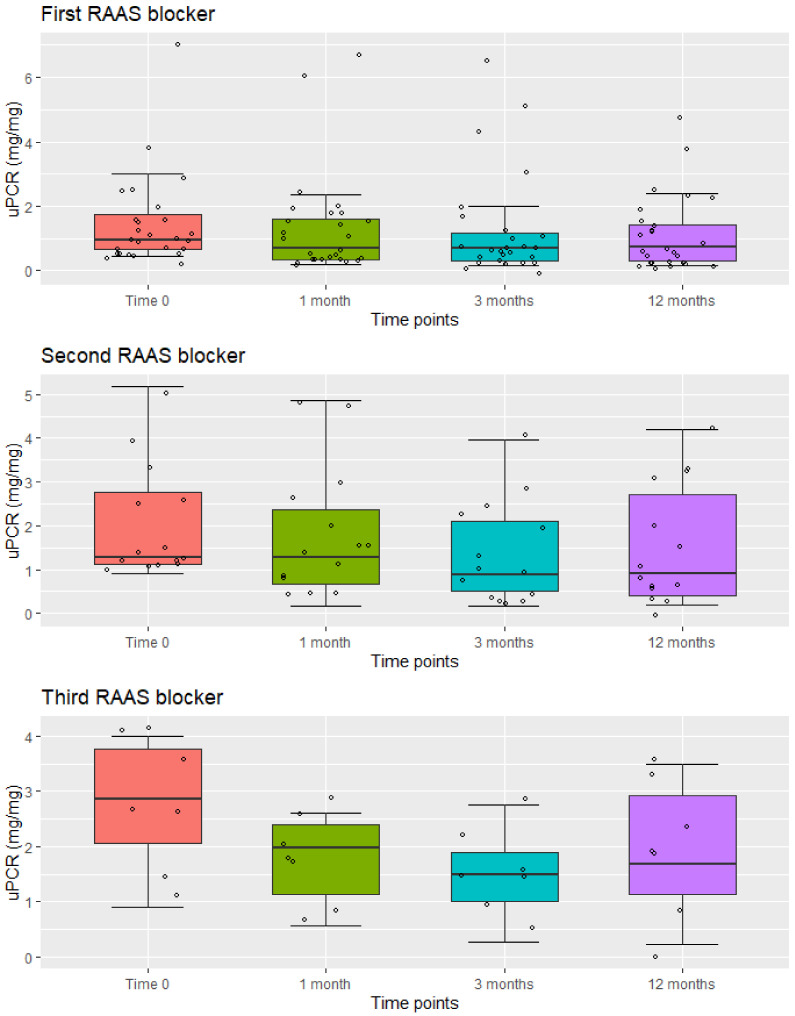
Boxplots of the distribution of uPCR values at different time points from the introduction of the first, second, and third RAAS blocker. The measured values are represented by circles.

In Table 2 uPCR values at baseline and at different time points from the introduction of first, second, and third RAAS blocker were reported. Before any treatment mean uPCR ratio was 1.46 ± 1.42 mg/mg. Figure 2 shows the boxplots of uPCR values.

Repeated Measure Anova analysis of log-transformed data shows that the reduction of uPCR values after Time 0 from the introduction of the first, second, and third drug was highly significant in all three cases (*p* values = 0.0016, 0.003, and 0.014, respectively). Figure 2, and pairwise *p* values in Table 2, show that the reduction was already significant after 1 or 3 months, while differences between individual time points after Time 0 were not significant.

Comparing uPCR values at Time 0 before the first drug to those at 12 months after the third drug (in the 7 patients who received a triple RAAS blockade), it was possible to observe that final values after all treatments were only slightly (and not significantly) higher than those before any treatment (means = 1.46 vs. 1.93, medians = 0.93 vs. 1.67). Three out of these 7 patients were males with X-linked AS (XLAS), 3 were females with autosomal recessive AS (ARAS), and 1 was a female with XLAS.

### 3.3. Renal Function

Table 3 shows mean eGFR values before and 1, 3, and 12 months after the introduction of a new RAAS blocker in our cohort of patients. The mean eGFR before treatment was 155.9 ± 49.3 mL/min/1.73 sm; no patient had chronic kidney damage before the start of therapy.

**Figure 3:**
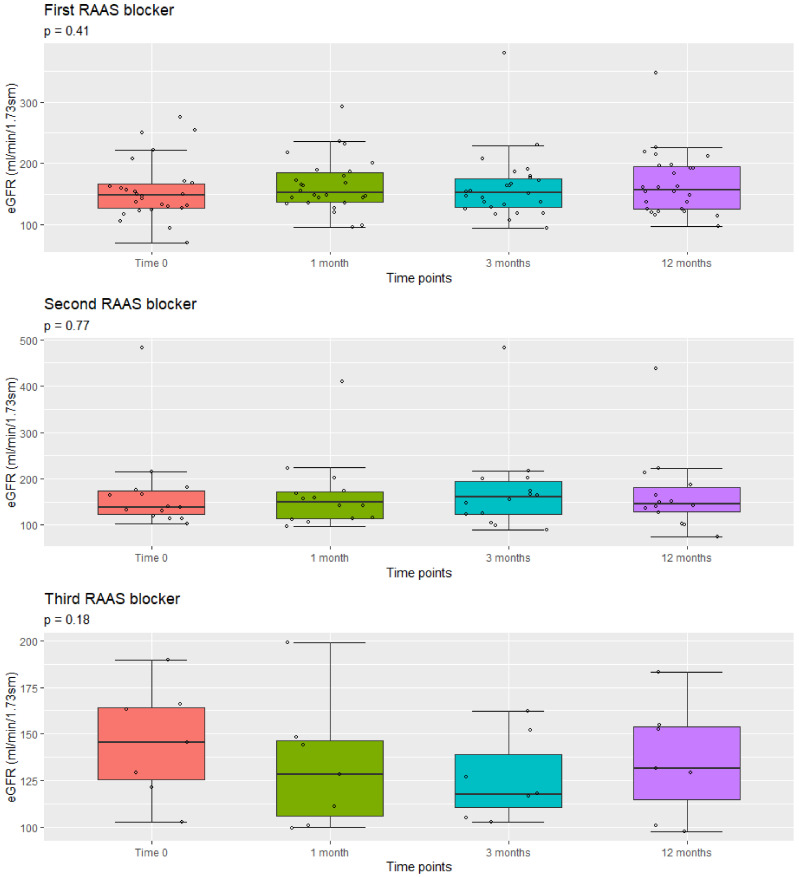
Boxplots of the distribution of eGFR values at different time points from the introduction of the first, second, and third RAAS blocker. All *p* values are from Repeated Measures Anova analysis of log-transformed data. The measured values are represented by circles.

No significant reduction in eGFR was observed during the follow-up period after the introduction of each new drug (*p* = 0.41, 0.77, 0.18, respectively). Mean eGFR was generally stable during follow-up (Figure 3). However, in a 15-year-old boy with XLAS, eGFR decreased to 74 mL/min/1.73sm, after 12 months of follow-up from the introduction of the second drug. Similarly, we did not observe a significant decline in eGFR values during the observation time of patients on triple RAAS blockade (*p* = 0.18 after 12 months).

### 3.4. Safety

No patient discontinued treatment within the first year of therapy in each group.

**Figure 4:**
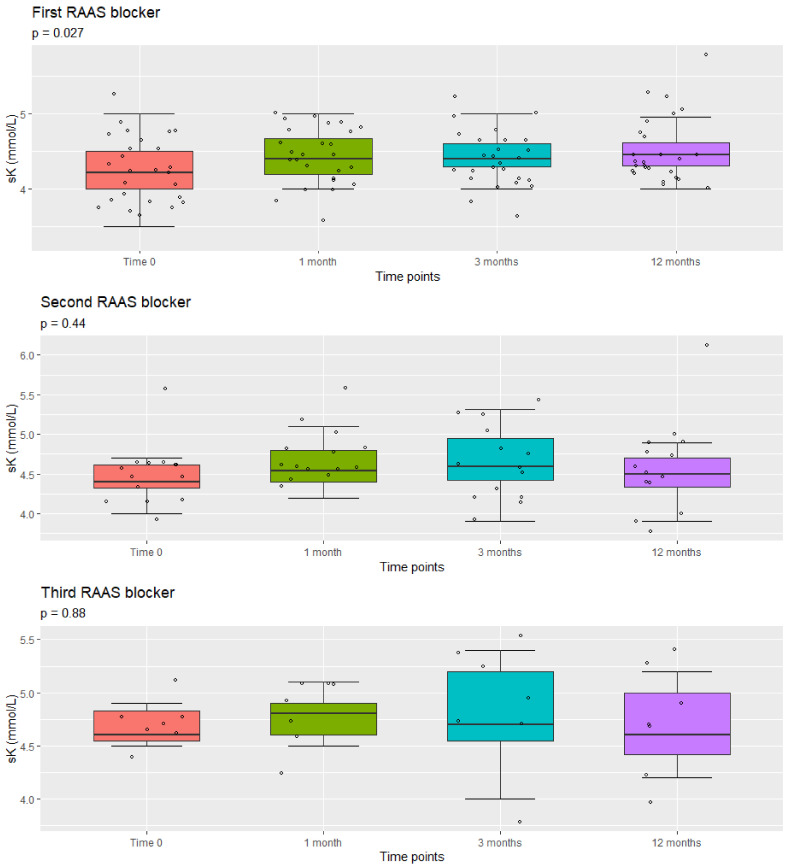
Boxplots of the distribution of sK values at different time points from the introduction of the first, second, and third RAAS blocker. All *p* values are from Repeated Measures Anova analysis.

Serum potassium levels (sK) were mostly normal (Figure 4, Table 4). A slightly significant increase in mean and median sK values was observed during the 1-year follow-up period after the introduction of the first RAAS blocker, although the values were still normal (less than 5.5 mmol/L) in all but one patient. This patient developed significant hyperkaliemia (K = 6.08 mmol/L) approximately 12 months after the start of the second RAAS blocker. This side effect resolved after ARB was stopped for 3 months and did not appear despite ARB reintroduction.

One patient developed gynecomastia and symptomatic hypotension and dropped out from the study 5 months after the beginning of SP as a third drug. He was already obese (weight 88.3 kg, height 169 cm, BMI 30.9 kg/sm) when he was started on the triple RAAS blockade. No one presented dry cough, headache, liver dysfunction, allergic reactions, cardiac arrhythmia, and muscle weakness, or any other side effect reported for these drugs. No life-threatening events were recorded.

## 4. Discussion

Our retrospective study suggests that sequential introduction of ACEi, ARB, and SP in pediatric proteinuric patients with AS study allows obtaining a progressive and synergic reduction of uPCR values, without changes of the glomerular function and with a good safety profile.

Many papers report that kidneys from patients with AS are characterized by a more fragile GBM. It could be disrupted even with normal intraglomerular pressure. RAAS blockers reduce the risk of GBM ruptures by decreasing the podocyte stress induced by intraglomerular pressure. In 2012, a retrospective observational study published by Gross et al. demonstrated that ACEi reduced proteinuria of AS patients and, consequently, delayed the onset of KF, improving life expectancy [15]. A subsequent study reported that losartan and enalapril had comparable efficacy in reducing proteinuria in AS children [8]. In 2016, Zhang et al. demonstrated that early and long-term treatment with both ACEi and ARB in children with AS was efficient and well-tolerated [10]. Thus far, dual RAAS blockade is part of the routine treatment of AS patients, despite no randomized clinical trial comparing single and double blockade [4,7].

Treatment with ACEi and/or ARBs leads to the reduction of serum aldosterone levels by blocking the conversion of angiotensin I to angiotensin II or inhibiting the effect of angiotensin II on specific receptors, respectively. The final effect is the lowering of serum aldosterone levels. However, it has been well described that serum aldosterone can rise to previous levels together with a parallel increase of the amount of proteinuria because of an up-regulation of enzymes such as chymase, which allows bypassing the block (the so-called “aldosterone escape” phenomenon) [6,16]. Thus, it has been hypothesized that the introduction of an aldosterone antagonist, leading to a triple RAAS blockade, should guarantee a stronger nephroprotective effect through also a reduction of proteinuria [11,12]. Moreover, aldosterone seems to play a central role in the progression of various glomerular proteinuric diseases, leading to KF as it promotes fibronectin production and induces fibrosis by stimulating the Transforming Growth Factor-β (TGF-β), a pro-fibrogenic cytokine, involved in the extracellular matrix synthesis [17,18,19,20]. In 2013 we showed a significant decrease of proteinuria, associated with a reduction of urinary TGF- β1 levels, in all patients with AS after beginning SP [13]. The results were confirmed during the follow-up with no serious side effects.

Taking these considerations into account, the results of our study suggest that sequential RAAS blockade therapy allows for safely achieving a decrease of proteinuria, within 12 months from the introduction of the additional pharmacological agent in pediatric patients with AS. We can speculate that the addition of SP to the dual RAAS blocking therapy could cause a reduction in proteinuria consequent to a more effective block of the RAAS system, despite the presence of the “aldosterone escape” effect due to the antagonist action on aldosterone receptors [11,12].

In addition, it is worth noting that the proteinuria-reducing effect appears early, being significant already 1 month after the introduction of the first, second, and third RAAS blocker.

The second and the third antiproteinuric drug were started at 2.17 ± 1.72 years and 5.55 ± 2.33 years after the introduction of the first and the second treatment, respectively. The natural history of patients with AS is characterized by isolated hematuria and subsequently by the appearance of proteinuria, and reduction of renal function up to the need for dialysis and transplantation. In our patients, the use of a triple RAAS blockade has allowed to freeze the progression of the disease during the early proteinuric phase for a long period. In fact, levels of uPCR 12 months after the introduction of the third RAAS blocker were not different from those before the start of the first drug (*p* = 0.47). The importance of these data is even greater if we consider that they are applied to a group of difficult-to-manage patients. Indeed, all our patients presented with an early appearance of proteinuria (10.55 ± 5.02 years) and had a severe form of AS (3 ARAS and 3 males XLAS), which per se have a worst prognosis [4]. Thus far, this type of therapeutic approach is to be considered the most effective, pending the results of the most recent studies on more modern therapies. In fact, more recently, studies have focused on new therapeutic options such as Bardoxolone [21], anti-microRNA-21 [22], stem cell-based therapies [23]. Furthermore, a recent innovation is the so-called “exon skipping therapy”, which, in mice with reduced expression of the α5 (IV) chain, has led to remarkable clinical and pathological improvements, including a higher expression of the α5 chain on glomerular and the tubular basement membrane, with better survival [24]. These data suggest that exon skipping may represent a promising therapeutic approach for treating severe male XLAS cases. At the moment, however, these therapies are not available in outpatient practice, pending solid data on their effectiveness.

In our study, the mean estimated eGFR remained stable during the first 12 months of treatment in all three groups. Furthermore, the eGFR did not change when comparing initial values, before the introduction of the first RAAS blocker, to final values at the end of each therapy (paired *p* values at the end of each block: 0.45, 0.76, 0.69, respectively), as in our previous study [13]. However, one of our patients developed a significant loss of glomerular function (eGFR = 74 mL/min/1.73 sm). We believe that it was probably related to the natural history of AS and we have not considered it as a side effect (*see Safety section*). In fact, his eGFR was in the low range of normality before starting the first drug (94.3 mL/min/1.73 sm). Moreover, patients in need of a second and a third drug must be considered in an advanced stage of the disease. On the other hand, our data seems to show high basal eGFR levels and a further increase of eGFR after the introduction of each drug, up to the hyperfiltration range. Anyway, it is worth noting that pediatric patients were considered to have normal filtration values up to 165 mL/min/1.73 sm [25], and that some authors have suggested that the threshold for glomerular hyperfiltration could vary up to 175 mL/min/1.73sm [26]. Furthermore, the eGFR did not change significantly after the introduction of each drug (paired *p*-values at the end of each block: 0.41, 0.77, and 0.18, respectively), even if a more evident reduction was noticeable after the introduction of the third drug.

No severe and life-treating side effects were recorded. We observed only a slightly significant increase in mean and median sK values during the 1-year follow-up period after the introduction of the first RAAS blocker, even if values were still normal (less than 5.5 mmol/L) in all but one patient. However, in this patient, sK values were only slightly increased, and he developed a significant hyperkaliemia (6.08 mmol/L) 12 months after double RAAS blockade, with no clinical consequences. Hyperkaliemia resolved after ARB was stopped. After a few months, ARB was re-introduced, with no hyperkaliemia. Only one patient developed gynecomastia and symptomatic hypotension and dropped out from the study. This patient was already obese before the onset of the therapy with SP, as a third drug. Anyway, he was considered for the analysis of patients on one and two drugs because the side effect appeared after the introduction of the third one.

The current study has limitations, such as small sample size and a retrospective design. The small number of patients is due to the rarity of AS and to the monocentric design of the study. Anyway, to our knowledge, this is one of the more representative monocentric cohorts reported. The retrospective design does not allow us to obtain long-term conclusions, although our data suggest the effectiveness of this kind of approach. In fact, other prospective studies have shown greater efficacy of RAAS blocker therapy if started at a very early stage of the disease [27]. If we consider these data and that our patients started therapy at a more advanced stage of the disease, we can hypothesize that the early initiation of the same therapeutic strategy could imply a longer time interval before starting the second and third drugs.

In conclusion, double and triple RAAS blockade is an effective, safe, and fast-acting therapeutic strategy to reduce proteinuria and freeze for a long period the progression of kidney damage in AS children. Nevertheless, we suggest carefully monitoring eGFR and Kaliemia during follow-up of children with AS being treated with ACEi, ARB, and SP. Further multicenter studies are necessary to confirm our findings.

## Figures and Tables

**Table 1:** Demographic and clinical data of patients at baseline. Proteinuria and creatinine clearance levels are presented as mean ± standard deviation (SD).

**Gender**	
Male	10 (38.5%)
Female	16 (61.5%)
**Mutation**	
absent	3
COL4A3 heterozygote	1
COL4A4 heterozygote	2
COL4A5 heterozygote	11
COL4A5 hemizygote	5
COL4A3 homozygote	1
COL4A4 homozygote	1
COL4A3 e COL4A5 dygenic	1
COL4A4 e COL4A5 dygenic	1
**Age at the onset of treatment (years)**	
10.55 ± 5.02	
**Proteinuria at onset (uPCR mg/mg)**	
1.46 ± 1.42	
**Estimated GFR at onset (mL/min/1.73sm)**	
155.93 ± 49.31	
**Serum K at onset (mmol/L)**	
4.29 ± 0.37	

**Table 2:** Distribution of uPCR values at different time points from the introduction of the first, second, and third RAAS blocker. Most data were significantly right-skewed. All *p* values are from Repeated Measures Anova analysis of log-transformed data.

uPCR (mg/mg)	Time 0 Mean ± SD Median	1 Month Mean ± SD Median	3 Months Mean ± SD Median	12 Months Mean ± SD Median	*p* Value	Pairwise *p* Values	
**1st** **drug** (*n* = 26)	1.46 ± 1.42 0.93	1.32 ± 1.57 0.69	1.27 ± 1.61 0.68	1.12 ± 1.13 0.75	0.0016	T0–1 m = 0.007 T0–3 m = 0.005 T0–12 m = 0.003	1 m–3 m = 0.29 1 m–12 m = 0.34 3 m–12 m = 0.78
**2nd** **drug** (*n* = 14)	2.02 ± 1.35 1.28	1.74 ± 1.52 1.27	1.36 ± 1.16 0.89	1.50 ± 1.35 0.90	0.003	T0–1 m = 0.068 T0–3 m = 0.006 T0–12 m = 0.007	1 m–3 m = 0.065 1 m–12 m = 0.27 3 m–12 m = 0.85
**3rd** **drug** (*n* = 7)	2.77 ± 1.20 2.87	1.74 ± 0.86 1.97	1.47 ± 0.82 1.50	1.93 ± 1.27 1.67	0.014	T0–1 m = 0.064 T0–3 m = 0.017 T0–12 m = 0.065	1 m–3 m = 0.18 1 m–12 m = 0.81 3 m–12 m = 0.31

**Table 3:** Distribution of eGFR values at different time points from the introduction of the first, second, and third RAAS blocker. Most data were significantly right-skewed. All *p* values are from Repeated Measures Anova analysis of log-transformed data.

eGFR (mL/min/1.73 sm)	Time 0 Mean ± SD Median	1 Month Mean ± SD Median	3 Months Mean ± SD Median	12 Months Mean ± SD Median	*p* Value
**1st** **drug** (*n* = 26)	155.93 ± 49.31 147.88	164.25 ± 44.28 152.05	160.62 ± 54.64 152.06	166.52 ± 52.52 157.03	0.41
**2nd** **drug** (*n* = 14)	169.78 ± 95.17 138.86	165.72 ± 79.31 149.67	175.01 ± 96.80 160.33	167.90 ± 87.93 146.40	0.77
**3rd** **drug** (*n* = 7)	145.33 ± 29.87 145.59	133.09 ± 35.05 128.23	126.08 ± 22.83 117.86	135.68 ± 30.58 131.70	0.18

**Table 4:** Distribution of sK values at different time points from the introduction of the first, second, and third RAAS blocker. All *p* values are from Repeated Measures Anova analysis.

sK (mmol/L)	Time 0 Mean ± SD Median	1 Month Mean ± SD Median	3 Months Mean ± SD Median	12 Months Mean ± SD Median	*p* Value
**1st** **drug** (*n* = 26)	4.29 ± 0.37 4.21	4.41 ± 0.38 4.40	4.40 ± 0.37 4.40	4.52 ± 0.37 4.45	0.027
**2nd** **drug** (*n* = 14)	4.51 ± 0.41 4.40	4.65 ± 0.40 4.53	4.65 ± 0.47 4.59	4.57 ± 0.52 4.50	0.44
**3rd** **drug** (*n* = 7)	4.68 ± 0.17 4.60	4.77 ± 0.23 4.80	4.80 ± 0.51 4.70	4.69 ± 0.38 4.60	0.88

## Data Availability

Data is contained within the article.
